# Strategies for knowledge mobilization by advanced practice nurses in three hospitals in Spain: a qualitative study

**DOI:** 10.1186/s12912-024-02095-5

**Published:** 2024-06-26

**Authors:** Concha Zaforteza-Lallemand, Ian Blanco-Mavillard, Sandra Pol-Castañeda, Carlos Javier Villafáfila-Gomila, Francisco Ferrer-Cruz, Miguel Ángel Rodríguez-Calero

**Affiliations:** 1 Department of Nursing and Physiotherapy, University of the Balearic Islands, Palma, Balearic Islands 07122 Spain; 2 Balearic Islands Health Services, Palma, Balearic Islands 07003 Spain; 3 Healthcare Implementation and Research Unit, Hospital Regional Universitario de Málaga, Avd. de Carlos Haya, 84. Málaga 29010, Málaga, 29010 Spain; 4 Hospital Son Llàtzer, Palma, Balearic Islands 07198 Spain; 5 Hospital Comarcal de Inca, Inca, Balearic Islands 07300 Spain; 6 CurES Research Group, Health Research Institute of the Balearic Islands (IdISBa), Palma, Balearic Islands 07120 Spain; 7 Quality Department, Torrecárdenas University Hospital, Almería, 04003 Spain

**Keywords:** Advanced practice nursing, Implementation science, Evidence-based practice, Practice guideline, Knowledge mobilization, Knowledge transfer, Knowledge translation

## Abstract

**Background:**

Evidence-based practice, in conjunction with optimum care quality, improves patients’ clinical outcomes. However, its implementation in daily clinical practice continues to present difficulties. The aim of this study was to identify the strategies applied by Advanced Practice Nurses (APNs) to foster adherence to clinical practice guideline recommendations.

**Methods:**

An exploratory qualitative study was conducted with six focus groups at three public hospitals belonging to the Balearic Islands Health Care Service (Spain). The study participants were 32 ward nurses and 5 advanced practice nurses working routinely with inpatients at these hospitals. The study was conducted from November 2020 to January 2021, using thematic analysis, based on the COREQ checklist.

**Results:**

Four major themes related to the facilitation process were identified either by RNs and APNs: the context of the project, APN contribution to nursing team management, healthcare provision on the ward, and the acquisition and application of knowledge.

**Conclusions:**

The APNs adapted their actions to the characteristics and needs of the local context, employing strategies aimed at improving teamwork, healthcare, and knowledge management. Each of these contributions enhanced the sustainability of the changes made.

**Supplementary Information:**

The online version contains supplementary material available at 10.1186/s12912-024-02095-5.

## What is already known about this topic

According to implementation science, multimodal strategies are context-tailored and use multiple approaches for successful behavioral change among healthcare providers. However, when a program is designed and implemented, little is known about how it is displayed in real life: which are the strategies and skills unfolded by implementation leaders, or why do providers choose (or not) to make things happen? For instance, there is limited available evidence regarding the mechanisms that change an a priori staff mistrust of a program by engaging it.

## What does this paper add

An intervention implementing APNs as facilitator agents of evidence-based practice (EBP) provides an improvement on patients’ health results. This paper underscores how the APNs tailored and managed to influence their peers’ adherence to EBP daily and face-to-face interaction. APNs’ multimodal strategies, skills and actions for practice change are well-described and acknowledged as valuable for changing behavior by peers and APNs themselves. In addition, the “making sense mechanism” triggered by a feedback strategy is identified as one of the leverages for trusting and valuing the intervention as positive for patients and the staff.

## What are the implications

This qualitative study helps health managers and implementation scientists to identify suitable profiles for the role of clinical champions and early adopters aimed at influencing their peers’ practice. Moreover, it contributes to monitoring change processes and program implementation, as the making sense mechanism occurrence points to a relevant unfolding of the initiative.

## Introduction

The implementation of evidence-based clinical practice (EBCP) [1, 2] and clinical practice guidelines (CPGs) [3] can present challenges to healthcare professionals, healthcare organizations, and the patients themselves [1]. Difficulties may arise due to the variable quality and nature of clinical evidence [5] and because of individual and contextual factors [2–4].

Many studies have reported on successful multimodal interventions to implement new evidence-based recommendations, such as those aimed at reducing peripherally-inserted venous catheter failure [5–7] even avoiding iatrogenic consequences and costs [8], or reducing pressure ulcer rates [9–12]. However, EBCP implementation is a multifaceted and complex phenomenon in continual and dynamic interaction between evidence, context, users, and facilitation [13, 14].

In addition, other studies have explored the role played by clinical champions [15] and Advanced Practice Nurses (APNs) as agents of change and enablers of evidence implementation [16–18]. Among other contributions, APNs provide problem-solving support and act as local agents of change [19], promoting awareness of and fidelity to best practices [14, 20].

In 2016 the Balearic Islands Health Service (Spain) conducted an internal evaluation on nursing adherence to evidence-based practice (EBP) which highlighted that there was room for improvement on that issue (unpublished report). The research team was invited to develop a project for enhancing EBP among nurses. Then the mixed-methods project “Moving Evidence into Practice by Advanced Practice Nurses in Hospitalization Wards” was launched. It was aimed at evaluating the impact made by APNs on hospital wards and patient outcomes in comparison with wards from which they were absent [21]. Briefly, five APNs and five support nurses were assigned to five hospital wards and given the introduction of new work dynamics for improving clinical outcomes. In particular, the APNs designed actions to implement CPGs, adapted to the local context and its characteristics. Although no specific regulation on APN is available in Spain, Regional Public Health Systems have developed local regulation and recognized some APN roles. The APNs participating in this project received formal appointments by the Balearic Islands Health Services. Selection was based on postgraduate training. All the participating APNs received specific training for the project in 9 competency areas, further explained in a previous publication [21, 22]. The CPGs adopted were “Care and maintenance of vascular access to reduce complications” [23] and “Prevention and treatment of pressure ulcers” [24], both in use in the Balearic Islands Health Service. Initial findings highlight that their presence improves adherence to CPGs and enhances patients’ clinical outcomes [22].

In this sense, Morena et al. [15], from a theoretical stand, highlight the need to empirically test how clinical implementation leaders (here APNs) impact provider behavior: what strategies do they deploy, and what mechanisms do they trigger to promote behavioral change? Our study findings could be of valuable assistance in further research efforts in this area [25], i.e. to foster the implementation and diffusion of evidence-based recommendations with the support of APNs. Therefore, as a part of the above-mentioned project, the objective of this part of the research was to identify the strategies applied by APNs to foster adherence to CPG recommendations.

## Methods

### Study design and setting

The study is an exploratory qualitative design, driven by a realist approach [26], in accordance with the i-PARIHS framework [14]. Under this theoretical lens, intended outcomes occur in a particular context in which certain mechanisms are triggered by how a program or policy is deployed.

The study was conducted in five wards in three Spanish State-funded acute care hospitals: Manacor Hospital, Inca County Hospital and Son Espases University Hospital that serve populations of 150,000, 134,000 and 330,000 inhabitants, respectively (see Table 1). The planned intervention has been briefly described above and published elsewhere [21, 22].

**Table 1:** Description of the complexity of the patients cared and number of beds by the healthcare organization

Hospital	Number of beds	Complexity	Medical services	Population
Son Espases University Hospital	1020	Tertiary care hospital	All medical and surgical services except burn units, some trasplantations and some paediatric highly complex surgeries	330,000
Manacor Hospital	224	Secondary care hospital	Internal medicine, general surgery, Orthopedic surgery, Urology, Pediatrics, Gynecology, Neurology, Pulmonology, Cardiology, Nephrology, Dermatology, Ophthalmology, Anesthesiology, Psychiatry, Radiology, Pathology, Intensive care, Emergency medicine, otorhynolaringology, oncology, gastroenterology, endocrinology, rehabilitation	150,000
Inca County Hospital	172	Secondary care hospital		134,000

### Participant selection and recruitment

Hospital ward nurses working routinely with inpatients at intervention units in the three hospitals were considered for enrolment in the study. The research team approached key informants to identify suitable participants, who in turn recruited others through a ‘snowball’ technique. Inclusion criteria were at least 2 years of experience as RN, being working as RN at any of the intervention units when the focus groups were conducted and had been working at any of the intervention unit for at least 6 months during the intervention period. Priority was given to including participants from a variety of professional backgrounds and career pathways. Exclusion criteria were not having experience as RN, not being working at the intervention units for 6 months more and not willing to participate in the study. 32 ward nurses were enrolled in an intentional sample and distributed into five focus groups. The sixth focus group was formed by the 5 APN (one for each intervention unit) (Table 2). The characteristics of APNs are also described in Suppl file 1.

**Table 2:** Participants and distribution into focus groups

Focus Group	Hospital	Hospital ward	Participants
1	Manacor Hospital	Medical ward	7 hospital nurses
2	Son Espases University Hospital	Medical ward	7 hospital nurses
3	Son Espases University Hospital	Surgical ward	8 hospital nurses
4	Inca County Hospital	Medical ward	4 hospital nurses
5	Manacor Hospital	Surgical ward	6 hospital nurses
6	Manacor Hospital, Son Espases University Hospital and Inca County Hospital	Medical and surgical wards	5 APNs

Participation was voluntary and without monetary compensation. Selection, recruitment and interviews ceased once data saturation had been achieved.

### Data collection

All the focus groups were moderated by 2 members of the research team (CZ-L and IB-M), except the one conducted at Inca County Hospital, moderated by (SP-C and MAR-C). The other researchers (CV-G, FF-C, JdP-G) occasionally participated as observers, completing a team of 3 researchers per focus group. The interview guide used for the groups was piloted in May 2020. The scheduled duration of each focus group was about 45 min. The sessions were held between September 2020 and January 2021 at locations and times convenient for the participants. Field notes were taken during and after each session. All sessions were audio-recorded and transcribed-professional transcription service was hired, with answers anonymized before the analysis. The participants spoke either in Spanish and Catalan and the transcriptor respected the speaker’s original language.

### Data analysis and rigor

A continuous and iterative thematic analysis was performed [27]. In the inductive phase, the transcripts were examined for units of meaning, concerning how facilitation by the APN took place in the hospital ward, and encoded. The resulting codes were then grouped into broader categories. Each focus group transcript was coded by two researchers (CZ-L and IB-M), working independently. During the deductive phase, the data were analyzed according to the elements proposed in the PARIHS theoretical framework and in the literature review.

Several triangulation strategies were employed, separately. The results were then compared and a triangulated coding tree was constructed and saturation agreed by three researchers (CZ-L, IB-M and SP-C) [28]. Two members of the research team (CZ-L and IB-M) discussed the data and the findings obtained. Another strategy applied to improve the experimental rigour was the development and recording of the researchers’ views on the methodological decisions taken during the study. An additional factor of importance is their dual status as clinicians and co-investigators [29]. Finally, the participants’ responses and the initial analysis were discussed by CF-Z and IB-M, who both have extensive experience in implementation science. Throughout this process, the Consolidated Criteria for Reporting Qualitative Studies (COREQ) checklist was used.

### Research team and reflexivity

The research team required detailed knowledge of the implementation process to properly interpret and contextualize the study data. Accordingly, four members of the research team (CZ-L, IB-M, MAR-C, SP-C), with previous experience in evidence implementation and social research, facilitated and conducted focus groups at the three participating hospitals. None had any previous relationship with any of the participants, which allowed them to establish a rapport and fostered an open and frank discussion. All of these researchers were healthcare professionals employed in the Balearic Islands Healthcare Service, except JdP-G, who teaches at the University of the Balearic Islands. The researchers are PhD, except CV-G, FF-C and SP-C who was a doctoral candidate at the moment of the study .CZ-L was the senior researcher in qualitative health research. IB-M and MAR-C were trained in qualitative research. CZ-L and SP-C are female; IB-M, MAR-C, CV-G, FF-C and JdP-G are male.

## Results

Four focus groups were conducted according to the mentioned methodology and guideline. All the invited participants were present in the focus groups, we did not register any withdrawal. In our analysis, four categories were identified: context (features of the context present at the beginning of the project), team management (how the APNs managed the team), healthcare management (how APNs managed the healthcare provision) and knowledge management. The coding tree, with these four categories, the codes and the verbatim transcripts, is shown in Supplementary Material 1. Supplementary Material 3 explains the acronyms used to identify who the participant was in each verbatim.

### Context

The role of APNs is relatively unknown within the Spanish health system. For this reason, especially at the beginning of the project, when there were still no tangible results reflecting the impact of their work, the relevance of these nurses was questioned by colleagues and managers (mistrust of new roles). For this reason, the APNs in our study felt continually obliged to justify their role and, at the same time, to differentiate it from that of the ward supervisor.

*Yes, you had to be constantly saying what your job was supposed to be. Because there are people* [referring to other colleagues and managers] *who thought this extra pay was nonsense, we could use this extra salary instead to have a back-up nurse and not for someone who’s there to, well, they didn’t know exactly what you were doing.* (FG 5, APN 1)

In addition, the staff members in each of the hospital wards considered their workload to be very demanding and therefore viewed the new projects presented with some distrust regarding their usefulness and the demands imposed (mistrust of new projects).

*As I said before, now what are they going to make us do? What are they* [referring to managers] *going to impose on us now? What control are they going to have over what we’re doing? And well, obviously it hasn’t been like that.* (FG 3 HUSE 1O, Nurse 5)

### Team management

The APNs employed three major facilitating strategies addressed to their colleagues, based on interpersonal relationships. Firstly, they sought to unite the team around a shared objective of excellence, via the implementation of EBCP (team cohesion).

*I think my team feels more like a team.* (FG 5, APN 3)

[The APN mimics herself asking her colleagues], “*What do you think needs to be improved?” and,”How can we do it together to make it better?” And that’s how it was.* (FG 5, APN 4)

Secondly, they sought to ensure a unity of discourse with the formal head of the ward unit, such that both would transmit the same message regarding healthcare excellence (consistency with institutional policy).

*Consistency in the messages given. The message that you give is the same, I mean, the professional one, if one day you’re not there, she is* [referring to the supervisor]; *if you’re not there, she is, and in the ward it’s the same message. And it’s just as strong when she says it as when I say it* (FG 5, APN 5).

Thirdly, mentoring was provided to newly recruited members of staff to ensure their rapid, effective integration into the work dynamics.

*That happened to me, too, the same as with her* [referring to a colleague who recounted a similar experience], *I had never worked in the ward, I’d always been in the operating room… And when I arrived, I was useless, because everyone is, at first, why not, it’s normal, you don’t know anything and there’s so much medication and you have to give a certain medication for a certain… It’s all very… Everything is always the same in the operating room, right? It doesn’t change much and, well, here, I was lost and, well, especially as far as the computer was concerned because in the operating room, we don’t usually use it so much, we don’t have treatment plans or anything, it’s more technical […] And the truth is that a leader like her* [referring to the APN of the ward], *because it helped me a lot, really a lot. Well, and all my workmates, obviously* (FG 2 HUSE 00, Nurse 3).

According to our analysis, concerning team management capabilities, the other ward staff members acknowledged that the APNs presented skills, knowledge and positive personal traits that facilitated change, generated confidence that this change was worthwhile and provided a valuable role model, encouraging them to follow this example. The healthcare professional traits cited were commitment, serenity, flexibility, patience, problem-solving capacity, discipline, and perseverance. Besides, the team members acknowledged that they had bought into the project due to APNs’ social skills and role-modelling traits.

Nurse 2: *It’s reassuring when the work gets to be stressful, she comes and says, “Come on, first this, then that…”.*

Nurse 4: *It’s a big relief* (FG 3 HUSE 1O).

Nurse 1: *I don’t know, but I think the same job might have been done by someone else and I don’t think it would have been as successful. Because she* [referring to the APN on her ward] *is really tireless, she’s helped us a lot.*

Nurse 4: *And she comes up behind you and says, “You haven’t seen this…”.*

Nurse 1: *And she chases after you, and she tells you, “This isn’t right…”.*

Nurse 4: *“I’d try to record it but if not, you do it, I don’t know what…”* (FG 2 HUSE 0O).

### Healthcare management

The APNs are instrumental in spurring the healthcare team to incorporate EBCP as a permanent aspect of daily practice. Among the actions applied to achieve this goal, the APN used to establish audits and evaluations of clinical results addressed to examine and analyze the evolution of health indicators. Based on these data, APNs implemented feedback rounds, informing the team about the impact on patient clinical status and the findings obtained in the audits. Finally, team meetings were organized, seeking consensus on the application of evidence, informing of news and developments and resolving any doubts/questions that may arise.

*I think that after having done the audits and as concerns the patients with ulcers and their skin care and everything else, yes, there have been improvements.* (FG 5, APN 5)

*When you take part in a feedback on the results they’ve achieved, that’s when they say, “Wow! Well, yes, we can really get somewhere.”* (FG 5, APN 4).

*Yes, because every day we have a meeting at half past nine in the morning, and of course, she* [referring to the APN of her ward] *is there from Monday to Friday, and we have a meeting about all of the patients; yes, of course, she always contributes, so you don’t have to worry about yesterday’s problems and there’s no-one you know, you always have her, and she helps us. That means there is continuity. Because when you’re doing rotating shifts you never have continuity, and she, well, she helps us to have…* (FG 4 HCIN HM1, Nurse 1).

In addition to these specific, instrumental actions, the APNs employed other, more diffuse strategies tailored to the culture of the wards, where nurses place great value on their proximity to patients and on maintaining good working relations. Taking these concerns into account, APNs used three strategies to facilitate the acceptance of practice change.

Firstly, they engaged, face-to-face, with the ward staff. Their permanent, physical presence on the ward, providing a fixed point of reference, meant that the other ward team members knew they could always rely on the APN for advice, support, knowledge, mediation, etc.

Nurse 6: *I think she does get quite involved, because with her I can compare two situations on one ward with another situation on another* [This nurse has worked in two wards with an APN].

Moderator: *The thing is, she has different skills and characteristics. The other way was perhaps dealing with more bureaucratic issues and this way is more that the work…*.

Nurse 6: [overlapping] *Right beside the patient.*

Nurse 3: *Right, whatever, for us it’s very important that the work is done beside the patient, it’s very important. It is not the same as with an APN who’s dealing with bureaucratic issues, of course, and it isn’t the same.*

Nurse 6: *Passing on data.*

Moderator: *Presence…*.

Nurse 2: *That’s right!*

Nurse 1: *For us it’s… when we’re on the morning shift and we see her* [referring to the APN on her ward] *it’s as if we were seeing, Wow! My God!*

Nurse 4: *Apart from the fact that it’s true, she’s always there […] And if she has to do it, for whatever reason, because she can see, because on top of everything, she can see it too, she’s ready to work with us. If she can see that we aren’t going to be able to do it, well, she will, she’s not afraid to muck in. The supervisors, though, they’re something else. (FG 3 HUSE 1O)*

Secondly, the APNs were sensitive and adaptative to the requirements of the environment: on the one hand, their work is cognitive and conceptual; on the other, they also participated in the manual and physical tasks of patient care during periods of maximum activity, alleviating the workload on the team. This contribution is another positive aspect of their physical presence on the ward.

The third strategy is about empathetic, respectful communication: other team members appreciate the inclusive communication style adopted, in which the APN is open to dialogue and does not impose a pre-determined view. This attitude generates security and confidence and helps avoid mistrust when clinical audits are conducted.

*You have to give it to her* [referring to the APN], *she’s done very well. Lots of tact. We never felt there was any type of imposition. She’s been really … when it came to, to focusing on how we should be doing it. She’s always been really friendly and we’ve never felt as if she was finding fault, which is what it might have seemed like at first with someone watching everything you do and what you don’t do. But it wasn’t like that. I mean, the APN did her job very well* (FG 3 HUSE 1O, Nurse 5).

### Knowledge management

The above-mentioned actions and strategies enabled APNs to act as effective intermediaries and distributors of knowledge. Face-to-face communication, mentoring and feedback were also influential in facilitating knowledge management.

In environments where actions to provide direct healthcare are predominant, it can be crucial to find the time to acquire new knowledge. In this respect, APNs facilitated access by organizing information and making it available to nurses through clinical sessions, the standardization of processes and the adaptation of evidence to local circumstances.

In addition, via feedback and team discussions, the APN triggered mechanisms to make sense of the indicators considered so that nurses associated the effort invested in changing their clinical practice with obtaining beneficial results for the patient and the organization. This assurance generated confidence in the value of the knowledge distributed by the APN and consolidated the sustainability of the changes effected.

Nurse 1: *I’ve definitely seen an improvement. Because in my day to day work I know more, thanks to what* [the APN] *passed on to us* [referring to feedback rounds and clinical data analysis].

Nurse 5: *Me too.*

Nurse 1: *Like updates on the latest treatment options… Even if you don’t try too hard* [to stay up-to-date], *you’ve got the latest knowledge, because there’s someone who does just that. Who does that job and makes sure you get what you need* (FG6 HMAN 3 A).

Nurse 6: *Well, it’s made work in the ward more organized. Before, it was like everyone did things however they saw fit, right?*

Moderator: *Let’s see, tell me about that.*

Nurse 1: *In treatment plans, for surgical patients, now we have a structured plan that you just have to go there* [referring to consulting the treatment plan]. *I mean, they’ve made it a little easier for us. Exactly, and organizing the work, that’s it…*.

Moderator: *Right.*

Nurse 6: *She doesn’t mind spending time… she’s made us a kind of handbook, like a handbook, and we know when a patient comes in, what we have to do, if it is for an operation or if they need to be admitted, because being admitted to the ward…*.

Nurse 3: *Yes, she’s set up a protocol, specific protocols.* (FG2 HUSE O0)

## Discussion

Our study shows that the APNs, deploy context-aware strategies whereby the facilitation process ensures the viability of the intervention, improves team cohesion, and mobilizes the best knowledge available. The mixed-methods approach described [21] would be an appropriate way to describe and evaluate complex strategies for change. In line with Astbury and Leeuw [30], we find that the evaluation of programs and policies requires methodologies with both conceptual and empirical components. In our case, clinical data (the empirical component) obtained from the audits reveal improvements in health outcomes with respect to pressure ulcers, on the one hand, and complications associated with venous catheters, on the other [22].

Mentoring, through their leadership and influence, is the main strategic action performed by the APNs, provided both to newly recruited nurses and to long-standing members of the team [31]. This activity, and the acknowledgment of its value, constitutes the mechanism underpinning the ward team’s confidence in the project. In this respect, Jagosh et al. [32] referred to the theory of social influence, whereby respected members of a community, who are directly involved in a given project, can impel others to take part and to support it. In addition, Morena et al. [15] emphasize the relevance of mentorship skills as a means of changing subjective norms and provider attitudes that shape healthcare delivery.

In another crucial capacity, APNs progressively incorporate innovations in healthcare, whilst respecting the individual and organizational characteristics of the context. Moreover, they create physical and social opportunities to enhance the integration of new recommendations into the system [31], such that they are not perceived by the nurses as a threat, for example, in terms of increased workload or responsibilities. The APNs also ensure that the change is not perceived as a vertical imposition, but rather as an opportunity to improve quality in healthcare practice [32].

Furthermore, APNs manage the provision and acquisition of new knowledge, making sure that it is accessible, clear and comprehensible, ensuring it is applied correctly by all concerned. A fundamental aspect of the facilitation role played by APNs is their adaptation to local needs of the recommendations set out in the CPGs, in order to optimize the effort invested by nurses in acquiring and applying new knowledge. Simultaneously, through multiple rounds of feedback [33], ward members see how clinical outcomes are improved when EBCP is applied. In other words, a notable response or mechanism is activated in healthcare professionals, namely the attribution of value to the effort invested in changing clinical practices.

According to realist theory, this mechanism arises from the underlying (social) entities, processes and structures that operate in the environment in question, and which explain why and how a strategy brings about certain changes in behavior. In the case we discuss, this mechanism operates through the perceptions, reasoning and actions of the participants, determining how they make use of the resources at their disposal to make the changes happen and to sustain them over time [33]. By applying elements of a realist theory-driven analysis, we can identify the conceptual logic of the intervention. In this case, the strategy of supplying clinical data feedback to professionals who are engaged with the project triggers the mechanism that gives meaning to the indicators measured in the audits. This mechanism, moreover, enables professionals to escape the danger of taskification [34], but rather to understand the meaning of the effort they are making and to sustain the change over time.

In accordance with realist theory [26], the future of program evaluation must advance from implementation theory towards theory-driven evaluation or program theory [35]. In the first case, it is assumed that the implementation of a program is the cause of change: thus, an evaluation based on the implementation theory will indicate that “program A brought about result B”. In the second case, it is assumed that programs are successful only if people choose to make them work: an evaluation drawn from program theory will affirm, “In context 1, the application of program A triggered mechanisms B and C, following which outcome D occurred”. Working from program theory allows us to open up the black box of how programs work in different contexts, focusing on the responses made by participants.

### Limitations

This study is subject to some limitations, which should be considered. Firstly, the results presented are strongly associated with the sociocultural, clinical, and organizational characteristics of the health and social systems prevailing in the Balearic Islands, and with the functions explicitly assigned and implicitly asserted by the nurses involved. In this study, moreover, focus groups were conducted with frontline nurses who participated in the areas addressed in the project; however, these nurses’ perceptions of the actions carried out at the organizational level may not accurately reflect current institutional policies, because the figure of the APN is not yet widely implemented in the Spanish healthcare system. On the other hand, a strong point of this study is that the consideration given to all the interventions made by the APNs provides a good basis for designing future interventions, better equipped to meet the needs of the diverse, changing contexts of the healthcare system. In addition, such a redesigned intervention would enhance the management of health teams, care provision and specialist knowledge.

## Conclusions

In conclusion, APNs play a pivotal role in adapting initial projects to the particularities and needs of the local context, making use of appropriate strategies to improve the management of personnel, healthcare provision and specialist knowledge. Through the lens of a realist approach, our study elucidates the transformative power of knowledge management, via results feedback to the team, triggers the mechanism for attributing value to the effort invested in change. This realization enhances the team’s confidence in innovative proposals and contributes to the sustainability of changes in clinical practice.

Our findings highlight a paradigm shift in the healthcare system, driven by APNs’ strategic actions that integrate context-aware innovations and mentorship, improving both health outcomes and the work environment. Finally, to understand how and why such programs succeed, it is not sufficient to describe their results; it is essential to unpack the black box and identify the underlying mechanisms that make team members decide to make the program work. This consideration should be addressed in greater detail in future organizational evaluation and research.

### Electronic supplementary material

Below is the link to the electronic supplementary material.

Supplementary Material 1

Supplementary Material 2

Supplementary Material 3

Supplementary Material 4

## Data Availability

The data that support the findings of this study are available on request from the corresponding author, IB-M, upon reasonable request.
