# Supplementary material for: Strategies for knowledge mobilization by advanced practice nurses in three hospitals in Spain: a qualitative study
Source: BMC Nurs. 2024 Jun 26;23:440. doi: 10.1186/s12912-024-02095-5 (PMC11202328; doi:10.1186/s12912-024-02095-5)
Supplement: Supplementary file 1 — Supplementary Material 1 [file 12912_2024_2095_MOESM1_ESM.docx]

**Supplementary material 1. The Consolidated Criteria for Reporting Qualitative Studies (COREQ) 32-item checklist for manuscript.**

**COREQ 32-item checklist for manuscript:**

| Domain 1: Research team and flexibility | | | Reported on page nº |
| --- | --- | --- | --- |
| Personal Characteristics | | | |
| 1 | Interviewer/facilitator | Which author/s conducted the focus group? | 7 |
| 2 | Credentials | What were the researcher’s credentials? | 8 |
| 3 | Occupation | What was their occupation at the time of the study? | 8 |
| 4 | Gender | Was the researcher male or female? | 8 |
| 5 | Experience and Training | What experience did the researcher have? | 8 |
| Relationship with participants | | | |
| 6 | Relationship established | Was a relationship established prior to study commencement? | 8 |
| 7 | Participant knowledge of the interviewer | What did the participants know about the researcher? Reasons for doing the research | N/A |
| 8 | Interviewer characteristics | What characteristics were reported about the interviewer? | 8 |
| Domain 2: Study design | | | |
| Theoretical framework | | | |
| 9 | Methodological orientation and theory | What methodological orientation was stated to underpin the study? | 6 |
| Participant selection | | | |
| 10 | Sampling | How were participants selected? purposive, convenience, consecutive, snowball | 7 |
| 11 | Method of approach | How were the participants approached? | 7 |
| 12 | Sample size | How many participants were in the study? | 7 |
| 13 | Non-participation | How many people refused to participate or dropped out? Reasons? | N/A |
| Setting | | | |
| 14 | Setting of data collection | Where was the data collected? | 7 |
| 15 | Presence of non-participants | Was anyone else present besides the participants and researchers? | N/A |
| 16 | Description of sample | What are the important characteristics of the sample? | 7 and 27 |
| Data collection | | | |
| 17 | Interview guide | Were questions, prompts, guides provided by the authors? Was it pilot tested? | 7 |
| 18 | Repeat interviews | Were repeat interviews carried out? If yes, how many? | N/A |
| 19 | Audio/visual recording | Did the research use audio or visual recording to collect data? | 7 |
| 20 | Field notes | Were field noted made during and/or after the focus group? | 7 |
| 21 | Duration | What was de duration of focus group? | 7 |
| 22 | Data saturation | Was data saturation discussed? | 7 |
| 23 | Transcripts returned | Were transcripts returned to participants for comment and/or correction? | N/A |
| Domain 3: Analysis and findings | | | |
| Data analysis | | | |
| 24 | Number of data coders | How many data coders coded the data? | 8 |
| 25 | Description of coding tree | Did authors provide a description of the coding tree? | 28 to 33 |
| 26 | Derivation of themes | Were themes identified in advance or derived from de data? | 8 |
| 27 | Software | What software, if applicable, was used to manage the data? | N/A |
| 28 | Participant checking | Did participants provide feedback on the findings? | N/A |
| Reporting | | | |
| 29 | Quotations presented | Were participant quotations presented to illustrate the findings? Was each quotation identified? | 28 to 33 |
| 30 | Data and findings consistent | Was there consistency between the data presented and the findings? | 28 to 33 |
| 31 | Clarity of major themes | Were major themes clearly presented in the findings? | 9 to 12 and 28 to 33 |
| 32 | Clarity of minor themes | Is there a description of diverse cases or discussion of minor themes? | 12 to 14 |
