# Supplementary material for: Strategies for knowledge mobilization by advanced practice nurses in three hospitals in Spain: a qualitative study
Source: BMC Nurs. 2024 Jun 26;23:440. doi: 10.1186/s12912-024-02095-5 (PMC11202328; doi:10.1186/s12912-024-02095-5)
Supplement: Supplementary file 2 — Supplementary Material 2 [file 12912_2024_2095_MOESM2_ESM.docx]

**Supplementary file 1. Guide for the focus group with advanced practice nurses**

Context-tailored multimodal strategies for effective knowledge mobilization by advanced practice nurses in three hospitals in Spain: A qualitative study

Zaforteza-Lallemand et al.

**Introduction**

As you know, the "Hospital Advanced Practice Nurse" project has been carried out in your unit. The project is being rolled out in phases in different services and units of the Balearic Islands Health Services. At the moment, an assessment of its impact and how it has been deployed is being carried out.

For this reason, we are interested in understanding your vision of the project and its impact. There are no right or wrong answers. With your contribution, you can help us understand how the project has been deployed and what its impact is.

The group meeting is being audio-recorded (you have just signed the informed consent), this allows us to analyze all relevant data without missing anything.

**Questions**

- **What has been your perception throughout the EPAH project?**
- **What have you done to deploy the project? How did you make it sustainable?**
- **What changed in your units/context? What things happened? Impact (Points of view are exposed from hospitals of different sizes)**
- **What barriers and facilitators for change did you find in each of your environments? What was the driving force for change?**
- Is there anything about the deployment of the projects that you want to add?
