# Supplementary material for: Strategies for knowledge mobilization by advanced practice nurses in three hospitals in Spain: a qualitative study
Source: BMC Nurs. 2024 Jun 26;23:440. doi: 10.1186/s12912-024-02095-5 (PMC11202328; doi:10.1186/s12912-024-02095-5)
Supplement: Supplementary file 4 — Supplementary Material 4 [file 12912_2024_2095_MOESM4_ESM.docx]

**SUPPLEMENTARY MATERIAL**

**Supplementary material 1. Characteristics of the Advanced Practice Nurses**

| Hospital | Participant | Post-graduate profile | Area of advanced practice |
| --- | --- | --- | --- |
| Hospital de Manacor | APN1 | Palliative care | Complex multi-chronic and palliative care |
| Hospital de Manacor | APN2 | Nursing Methodology | Older people with trauma diseases |
| Hospital Comarcal de Inca | APN3 | Patient safety | Complex multi-chronic and palliative care |
| Hospital Universitario de Son Espases | APN4 | Research and innovation in health | Oncology |
| Hospital Universitario de Son Espases | APN5 | Patient safety | Older people with trauma diseases |

APN: Advanced practice nurses

**Supplementary material 2.** Themes, codes and verbatims of study.

| Themes | Codes | Verbatim |
| --- | --- | --- |
| **Project context**  Two features common to the hospital wards included in the study, and present at the beginning of the project. | Mistrust of new projects (care burden) | *As I said before, now what are they going to make us do? What are they going to impose on us now? What control are they going to have over what we’re doing? And well, obviously it hasn’t been like that.* (FG 3 HUSE 1O, Nurse 5) |
|  | Mistrust of new roles | *Yes, you had to be constantly saying what your job was supposed to be. Because there are people who thought this extra pay was nonsense, we could use this extra salary instead to have a back-up nurse and not for someone who’s there to, well, they didn’t know exactly what you were doing.* (FG 5, APN 1) |
| **Ward team management**  The set of actions and personal capacities applied by the APNs to enhance/facilitate the team’s work, integrating the changes made and minimising the barriers of the context. The actions taken belong to clearly identifiable strategies. The APNs’ capabilities reflect personal behavioural characteristics that colleagues and the APNs themselves recognise as appropriate for making changes to the team’s work practices. | **What is done (actions)**  Team cohesion | *I think my team feels more like a team.* (FG 5, APN 3)  [The APN mimics herself asking her colleagues], “*What do you think needs to be improved?” and,”How can we do it together to make it better?” And that’s how it was.* (FG 5, APN 4) |
|  | **What is done (actions)**  Consistency with the official line | *Consistency in the messages given. The message that you give is the same, I mean, the professional one, if one day you’re not there, she is [referring to the supervisor]; if you’re not there, she is, and in the ward it’s the same message. And it’s just as strong when she says it as when I say it* (FG 5, APN 5). |
|  | **What is done (actions**  Mentoring new members of staff | *That happened to me, too, the same as with her* [referring to a colleague who recounted a similar experience], *I had never worked in the ward, I’d always been in the operating room... And when I arrived, I was useless, because everyone is, at first, why not, it’s normal, you don’t know anything and there’s so much medication and you have to give a certain medication for a certain... It’s all very… Everything is always the same in the operating room, right? It doesn’t change much and, well, here, I was lost and, well, especially as far as the computer was concerned because in the operating room, we don’t usually use it so much, we don’t have treatment plans or anything, it’s more technical [...] And the truth is that a leader like her* [referring to the APN of the ward], *because it helped me a lot, really a lot. Well, and all my workmates, obviously* (FG 2 HUSE 00, Nurse 3)  *A really important support for those of us who come in as relief staff, when we don’t know where everything is, you know?* (FG 1 HMAN 2B, Nurse 1) |
|  | **How it is done**: Via qualities such as commitment, serenity, flexibility, patience, problem-solving capability, discipline and perseverance**.** | *She* [referring to the APN of her ward] *is so engaged, and little by little she’s made us feel comfortable about the project. (FG 3 HUSE 1O, Nurse 5)*  *Nurse 2: It’s reassuring when the work gets to be stressful, she comes and says, “Come on, first this, then that…”.*  *Nurse 4: It’s a big relief (FG 3 HUSE 1O)*  *She never gets angry about anything. (FG 2 HUSE 0O, nurse 4)*  *Nurse 1: I don’t know, but I think the same job might have been done by someone else and I don’t think it would have been as successful. Because she* [referring to the APN on her ward] *is really tireless, she’s helped us a lot.*  *Nurse 4: And she comes up behind you and says, “You haven’t seen this...”.*  *Nurse 1: And she chases after you, and she tells you, “This isn’t right...”.*  *Nurse 4: “I’d try to record it but if not, you do it, I don’t know what…” (FG 2 HUSE 0O)* |
| **Treatment management**  The specific actions that the APN deploys at the patient’s bedside or at the organisation level to facilitate the incorporation of new knowledge into the team’s daily practice. Also, how these actions are taken, considering the unit’s culture and values, to reinforce the acceptance of change. | **What is done (actions)**  Audits and the evaluation of clinical results. | *I think that after having done the audits and as concerns the patients with ulcers and their skin care and everything else, yes, there have been improvements.* (FG 5, APN 5) |
|  | **What is done (actions)**  Feedback*.* | *When you take part in a feedback on the results they’ve achieved, that’s when they say, “Wow! Well, yes, we can really get somewhere.”* (FG 5, APN 4) |
|  | **What is done (actions)**  Team meetings. | *Yes, because every day we have a meeting at half past nine in the morning, and of course, she* [referring to the APN of her ward] *is there from Monday to Friday, and we have a meeting about all of the patients; yes, of course, she always contributes, so you don’t have to worry about yesterday’s problems and there’s no-one you know, you always have her, and she helps us. That means there is continuity. Because when you’re doing rotating shifts you never have continuity, and she, well, she helps us to have...* (FG 4 HCIN HM1, Nurse 1) |
|  | **How it is done**:  Physical presence.  Sensitivity and adaptation to local values. | *Nurse 6: I think she does get quite involved, because with her I can compare two situations on one ward with another situation on another* [This nurse has worked in two wards with an APN]  *Moderator: The thing is, she has different skills and characteristics. The other way was perhaps dealing with more bureaucratic issues and this way is more that the work...*  *Nurse 6:* [overlapping] *Right beside the patient.*  *Nurse 3: Right, whatever, for us it’s very important that the work is done beside the patient, it’s very important. It is not the same as with an APN who’s dealing with bureaucratic issues, of course, and it isn’t the same.*  *Nurse 6: Passing on data.*  *Moderator: Presence.*  *Nurse 2: That’s right!*  *Nurse 1: For us it’s… when we’re on the morning shift and we see her* [referring to the APN on her ward] *it’s as if we were seeing, Wow! My God!*  *Nurse 4: Apart from the fact that it’s true, she’s always there [...] And if she has to do it, for whatever reason, because she can see, because on top of everything, she can see it too, she’s ready to work with us. If she can see that we aren’t going to be able to do it, well, she will, she’s not afraid to muck in. The supervisors, though, they’re something else. (FG 3 HUSE 1O)*  …*she’s always there, always* (FG1 HMAN 2B) |
|  | **How it is done**:  Empathic, respectful communication | *You have to give it to her, she’s done very well. Lots of tact. We never felt there was any type of imposition. She’s been really … when it came to, to focusing on how we should be doing it. She’s always been really friendly and we’ve never felt as if she was finding fault, which is what it might have seemed like at first with someone watching everything you do and what you don’t do. But it wasn’t like that. I mean, the APN did her job very well (FG 3 HUSE 1O, Nurse 5)* |
| **Management of knowledge**  Actions and strategies applied by the APN to supply and implement the knowledge required for the proposed change. | **What is done**: APNs organise and make knowledge accessible through clinical sessions, by standardising processes, and by adaptation to local circumstances. | *Nurse 1: I’ve definitely seen an improvement. Because in my day to day work I know more, thanks to what [the APN] passed on to us.*  *Nurse 5: Me too.*  *Nurse 1: Like updates on the latest treatment options... Even if you don’t try too hard* [to stay up-to-date], *you’ve got the latest knowledge, because there’s someone who does just that. Who does that job and makes sure you get what you need (FG6 HMAN 3A)*  *Nurse 6: Well, it’s made work in the ward more organised. Before, it was like everyone did things however they saw fit, right?*  *Moderator: Let’s see, tell me about that.*  *Nurse 1: In treatment plans, for surgical patients, now we have a structured plan that you just have to go there* [referring to consulting the treatment plan]. *I mean, they’ve made it a little easier for us. Exactly, and organising the work, that’s it…*  *Moderator: Right.*  *Nurse 6: She doesn’t mind spending time... she’s made us a kind of handbook, like a handbook, and we know when a patient comes in, what we have to do, if it is for an operation or if they need to be admitted, because being admitted to the ward...*  *Nurse 3: Yes, she’s set up a protocol, specific protocols. (FG2 HUSE O0)* |
|  | **What tools are used**: team management strategies are applied to facilitate change, together with aspects of knowledge management such as mentoring colleagues, availability, feedback. |  |
|  | **What mechanisms are activated (triggers):**  Making sense of change: the nurses acknowledge the improvements obtained in the indicators. | *Nurse 4: They are recorded* [referring to the use of peripheral venous lines]. *She takes them out. So, then we can see why there are infections because, you know, the point is that it’s important to take the catheter out, and to know when it was inserted and when it was taken out, how many days... Those things, maybe you put them in and take them out...*  *Nurse 1: And it is just the fact of saying, it’s that we do it this way and we have to record it so as to have control, and that has turned out very well... (FG2 HUSE O0)* |

**Supplementary material 3: how to interpret verbatim authorship**

Verbatim is labelled at the bottom of each paragraph indicating who was speaking and which institution did the participant belong to. The table below addresses the acronyms’ meanings.

|  | **Acronym** | **Meaning** |
| --- | --- | --- |
|  | FG | Focus group |
| Who was speaking | APN | Advanced practice nurse |
|  | Nurse | Registered Nurse (staff) |
| Which organization did the nurse belong to | HUSE | Hospital Universitario Son Espases (Son Espases University Hospital) |
|  | HCIN | Hospital Comarcal d’Inca (Inca County Hospital) |
|  | HMAN | Hospital de Manacor (Manacor County Hospital) |
| Which ward did the nurse belong to | 0O | Ward (HUSE) |
|  | 1O | Ward (HUSE) |
|  | 3A | Ward (HMAN) |
|  | HM1 | Ward (HCIN) |

Examples:

- FG 5, APN 1: focus group number 5, Advanced Practice Nurse number 1
- FG 3, HUSE 1O, Nurse 5: focus group number 3, Son Espases University Hospital, ward 1 O.
